# Enhanced lymphangiogenesis in the left lateral segment of a biopsied liver during portoenterostomy for biliary atresia

**DOI:** 10.1007/s00383-024-05845-3

**Published:** 2024-10-04

**Authors:** Yudai Tsuruno, Koshiro Sugita, Toshihiro Muraji, Ryuta Masuya, Toshio Harumatsu, Keisuke Yano, Shun Onishi, Takafumi Kawano, Chihiro Ichikawa, Haruo Ohtani, Yuko Bitoh, Satoshi Ieiri

**Affiliations:** 1 Department of Pediatric Surgery, Research Field in Medical and Health Sciences, Medical and Dental Area, Research and Education Assembly, Kagoshima University, Kagoshima, Japan; 2 Department of Pediatric Surgery, Kakogawa Central City Hospital, Kakogawa, Japan; 3 Division of Pediatric Surgery, Department of Surgery, Kobe University Graduate School of Medicine, Kobe, Japan; 4 Division of Gastrointestinal, Endocrine and Pediatric Surgery, Department of Surgery, Faculty of Medicine, University of Miyazaki, Miyazaki, Japan; 5 Department of Pathology, Kakogawa Central City Hospital, Kakogawa, Japan; 6 Department of Pathology, Mito Saiseikai General Hospital, Mito, Japan

**Keywords:** Biliary atresia, Biopsy, Lymphangiogenesis, Portal vein

## Abstract

**Purpose:**

We investigate the histopathology of the portal vein branches and lymphatic vessels to elucidate the mechanism of atrophy of the left lateral segment (LLS) of the liver in biliary atresia (BA).

**Methods:**

LLS and right anterior segment (RAS) liver biopsy samples obtained during Kasai portoenterostomy (KPE) from ten consecutive patients with BA underwent histopathological investigation of the portal vein and lymphatic vessels using double chromogenic immunostaining for CD31/D2-40 and the hepatitis-like findings (HLF) score. Each parameter and clinical data were compared between prognostic groups.

**Results:**

HLF scores in the LLS were always higher than those in the RAS. There was no difference in portal vein and lymphatic vascular morphology, whereas the number of lymphatic vessels was correlated with the fibrotic area of all specimen areas. Left-to-right ratio of the number of lymphatic vessels was correlated with the age at KPE (*r* = 0.784, *p* = 0.007) and the pre-KPE CRP value (*r* = 0.723, *p* = 0.018).

**Conclusions:**

Lymphangiogenesis on the LLS compared to the RAS was significantly correlated with the degree of fibrosis and the age at KPE. Further investigation is warranted to clarify the causes of LLS atrophy and lymphangiogenesis relevant to immune dysregulation.

## Introduction

The cause of biliary atresia (BA) remains unclear, although viral, developmental, and immune-mediated mechanisms have been proposed as possible causes. The BA liver removed at the time of liver transplantation frequently presents with atrophy of the left lateral segment (LLS) compared to the other segments [1]. However, the cause of this heterogeneous atrophy has not yet been clarified. Masuya [2] reported that the number of small-diameter portal vein (PV) branches is significantly higher in liver biopsy specimens obtained during Kasai portoenterostomy (KPE), and their diameters were not significantly correlated with the degree of fibrosis. Unraveling the mechanism of this heterogeneous atrophy of the BA liver could provide clues for understanding the etiopathogenesis of BA. As it is well recognized that any portal blood flow impairment leads to progressive parenchymal atrophy, we investigated the histopathology of the vessels to compare the left and right segments of the BA liver.

## Patients and methods

### Study design and population

Ten patients with BA who underwent Kasai portoenterostomy (KPE) at our institution between February 2018 and June 2023 were consecutively enrolled in this study. All patients underwent liver biopsies of the left lateral segment (LLS) and right anterior segment (RAS) during KPE. The morphology of the portal vein and lymphatic vessels in liver biopsy specimens was histopathologically investigated to determine any differences between LLS and RAS. The clinical significance of these differences was evaluated from a prognostic perspective.

### Patient background

The patient backgrounds included age at surgery and preoperative blood tests, such as white blood cell counts (WBC), C-reactive protein (CRP), direct bilirubin (d-bil), alanine aminotransferase (ALT), and γ-glutamyl transpeptidase (γ-GTP).

### Histopathological staining

The liver specimens were fixed in formalin and embedded in paraffin (FFPE). Paraffin blocks were sliced into 4 μm-thick sections and stained after deparaffinization. The stained left and right liver tissues were placed on a single slide. Double chromogenic immunostaining for CD31 + podoplanin (D2-40) was performed to distinguish between portal veins and lymphatic vessels. The details of this staining process have been described in our previous study [2].

### Histopathological assessment

The histopathological assessment was performed by a pediatric surgeon using the Olympus cellSens software (OLYMPUS Corporation, Tokyo, Japan) in collaboration with a pathologist (C.I.). Three adjacent lobules with portal tracts were randomly selected for double immunohistochemical staining for CD31 and podoplanin (D2-40) specimens, and the number and diameter of the portal veins stained with CD31 and the number of lymphatic vessels with or without lumens stained with D2-40. The fibrotic portal tracts were evaluated using Elastica-Masson Trichrome (EMT) stained specimens in the same measurement range as above. Hepatitis-like findings (HLF) were analyzed according to a previous study [3]. Briefly, HLF includes three histological features of the BA liver: hepatocyte multinuclear change (≥ 4 nuclei/cell), ballooning, and acidophilic bodies, each of which was semi-quantitatively rated as 0 for nil, 1 for scanty, and 2 for abundant. The HLF is expressed as the sum of scores, ranging from 0 to 6. Examples of these changes and scoring systems are shown in the figures from a previous study. The analyzed tissue evaluations were re-evaluated by two other pediatric surgeons to confirm that there were no inconsistencies in the results.

### Statistical analyses

Two-group comparisons for continuous variables were performed using the Mann–Whitney *U* test, and the values are presented as the median and interquartile range. A correlation analysis was performed using a non-parametric test with the Spearman’s rank correlation coefficient. All results were considered to be statistically significant when the *p*-value was < 0.05. All statistical analyses were performed using EZR (Saitama Medical Center, Jichi Medical University, Saitama, Japan), a graphical user interface for R (The R Foundation for Statistical Computing, Vienna, Austria). More precisely, it is a modified version of the R commander designed to add statistical functions frequently used in biostatistics [4].

### Ethical approval

This study was performed in accordance with the Ethical Guidelines for Medical and Health Research Involving Human Subjects by the Ministry of Health, Labour and Welfare of Japan in 2014 and it was in compliance with the 1964 Declaration of Helsinki, revised in 2013. This study was approved by the local ethics committee of our institution (Grant number 210329).

## Results

### Results of the patients’ background characteristics and preoperative blood tests

Clinical data for each patient are shown in Table 1. The median age at KPE was 62.7 days (range 31–108 days). Six patients were native liver survivors, and the remaining four patients underwent liver transplantation. All parameters of the preoperative blood tests were not significantly different between the LT group and the NLS group [CRP value, LT group vs. NLS group: 0.13 (0.05–0.22) vs. 0.12 (0.07–0.58), *p* = 1.00] [WBC, LT group vs. NLS group: 11,210 (8890–14850) vs. 10,550 (5800–13090), *p* = 0.762] [d-bil, LT group vs. NLS group: 4.85 (2.6–6.7) vs. 3.95 (2.5–8.0), *p* = 0.915] [ALT, LT group vs. NLS group: 65.0 (29.0–146) vs. 54.5 (20.0–104), *p* = 0.762] [γ-GTP, LT group vs. NLS group: 328 (111–547) vs. 344 (229–1094), *p* = 0.61].

**Table 1:** Patients’ background characteristics and preoperative blood tests

No	Sex	KPE (Days)	Type	D-Bil (mg/dL)	γ-GT (U/L)	CRP (mg/dL)	Prognosis	Indication for LT
1	F	65	III-b1-ν	6.8	229	0.58	NLS	–
2	M	46	III -a1-ν	2.5	308	0.16	NLS	–
3	M	61	III -b1-μ	5.4	547	0.10	LT	Cholangitis
4	F	98	II-a2-o	8.0	1094	0.14	NLS	–
5	M	58	III-b1-μ	4.9	903	0.10	NLS	–
6	F	73	III-b1-μ	2.5	344	0.08	NLS	–
7	F	46	III-b2-μ	2.6	352	0.05	LT	Persistent jaundice
8	F	41	III-b2-ν	4.3	111	0.15	LT	Persistent jaundice
9	M	108	III-b1-ν	6.7	304	0.22	LT	Persistent jaundice
10	F	31	III-b1-μ	3.0	343	0.07	NLS	–
Median	–	62.7	–	4.6	343	0.13	–	–

### Portal vein morphology

**Fig. 1:**
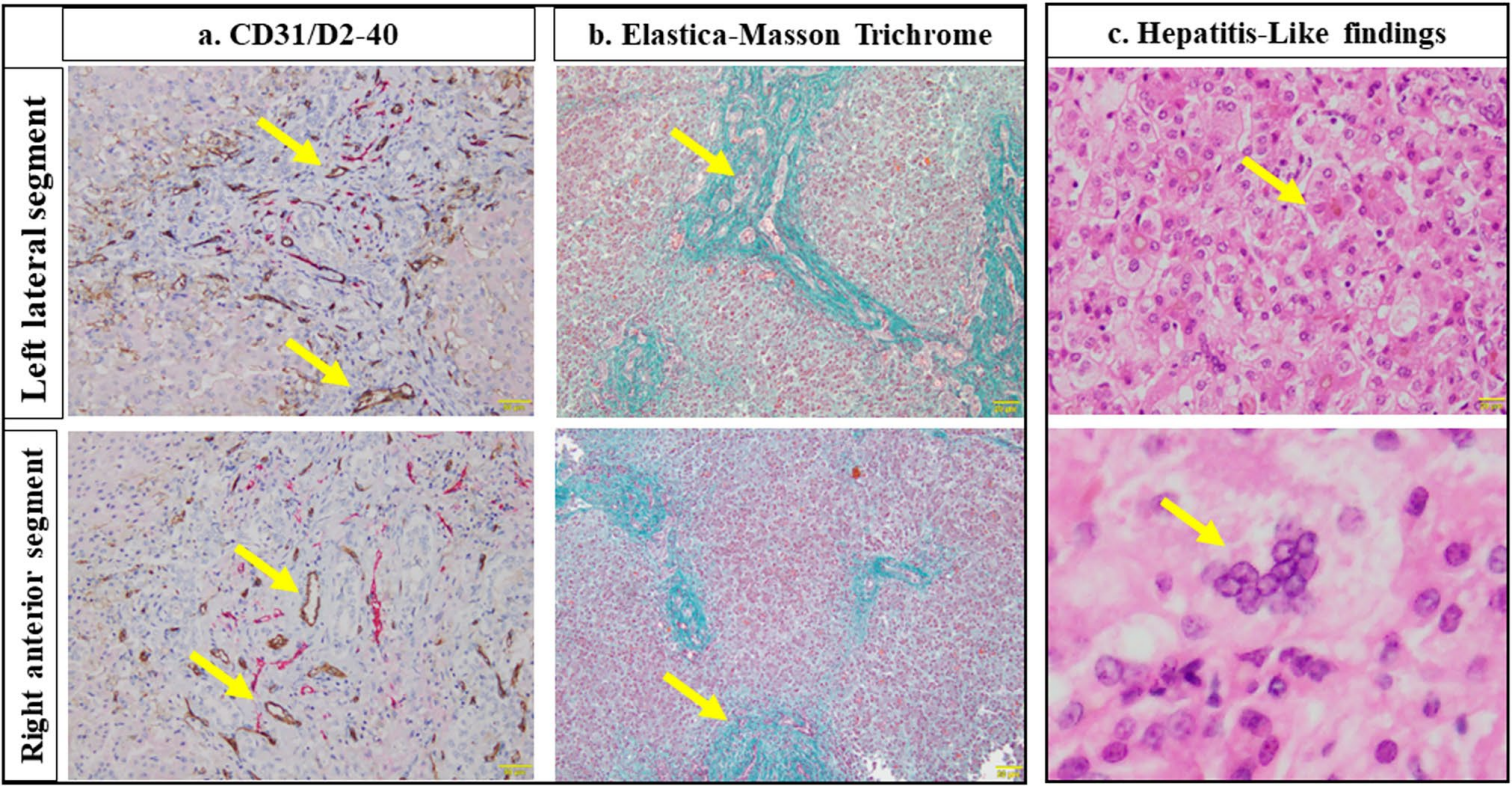
Representative histopathological findings of biopsy liver specimens. **a** CD31/D2-40; yellow arrow, portal vein. **b** Elastica-Masson Trichrome; yellow arrow, fibrotic area. **c** Hepatitis-Like findings; yellow arrow (top row), acidophilic body; yellow arrow (bottom row), hepatocyte multinuclear change

Representative histopathological findings of the biopsy liver specimens are shown in Fig. 1. Figure 1a shows double immunohistochemistry for CD31 and D2-40 counterstained with hematoxylin while Fig. 1b shows EMT staining.

There were no significant differences in the number of portal vein branches, portal vein diameters, and fibrosis area at KPE between the left and right liver specimens [number of portal vein branches, left vs. right: 28 (21–58) n/mm^2^ vs. 25 (17–34) n/mm^2^, *p* = 0.405] [portal vein diameters, left vs. right: 6.7 (6.4–8.0) μm/n vs. 7.1 (5.8–7.9) μm/n, *p* = 0.705] [fibrosis area, left vs. right: 22.8 (18.8–27.2) % vs. 19.7 (14.4–22.8) %, *p* = 0.326].

There was no significant correlation between the age at KPE and the left-to-right ratio of the number of portal vein branches, portal vein diameters, or fibrosis area (age at KPE vs. ratio of number of portal vein branches [left/right]: *r* = 0.322, *p* = 0.364) [age at KPE vs. ratio of portal vein diameters (left/right): *r* = − 0.073, *p* = 0.841] [age at KPE vs. ratio of fibrotic area (left/right): *r* = 0.286, *p* = 0.424].

No significant difference in the portal vein morphology was observed between LLS and RAS, regardless of KPE age.

### Hepatitis-like findings

**Fig. 2:**
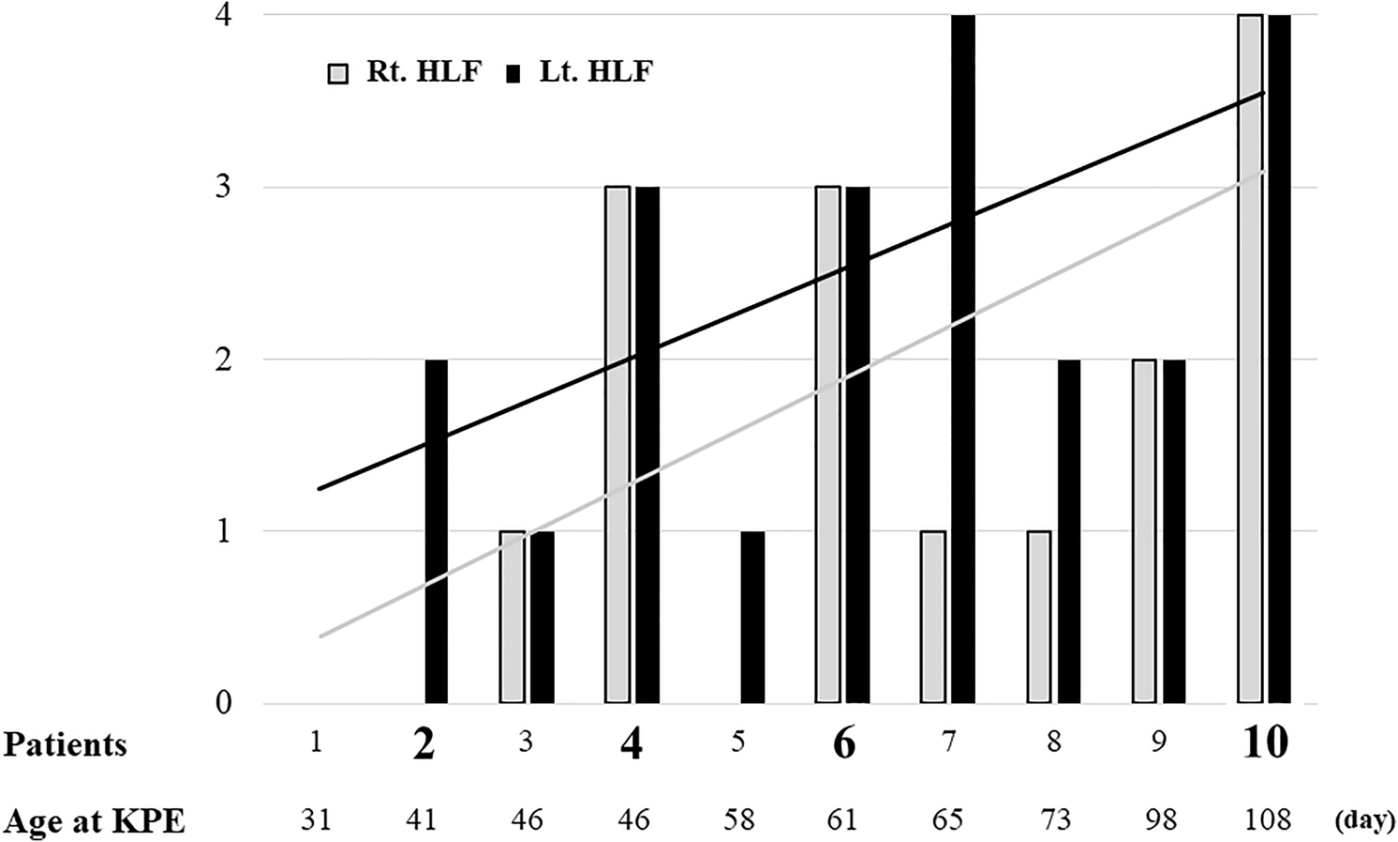
Hepatitis-like findings in the left and right liver in each patient. Rt. HLF, light color; Lt. HLF, dark color. Patients; large bold, liver transplantation. Age at KPE (day); KPE, Kasai portoenterostomy

Representative HLF of the biopsy liver specimens are shown in Fig. 1c. The HLF scores in the LLS and RAS of each patient are shown in Fig. 2. There tended to be a positive correlation between the HLF score and age at KPE, although the difference was not statistically significant, for LLS (*r* = 0.590, *p* = 0.072) and RAS (*r* = 0.622, *p* = 0.055). The LLS scores were always higher than or equal to those of the RAS, and there was no single case where LLS was lower.

### Lymphatic vessels morphology

**Fig. 3:**
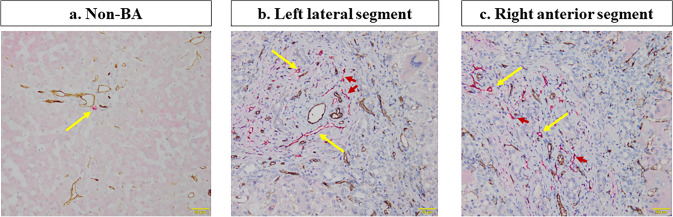
Representative findings of the lymphatic vessel morphology in biopsy liver specimens. **a** Non-BA, 2 years-old patient with choledochal cyst; **b** BA, left lateral segment; **c** BA, right anterior segment. Yellow arrow, lymphatic vessels with lumen; Red arrow, lymphatic vessels without lumen

**Fig. 4:**
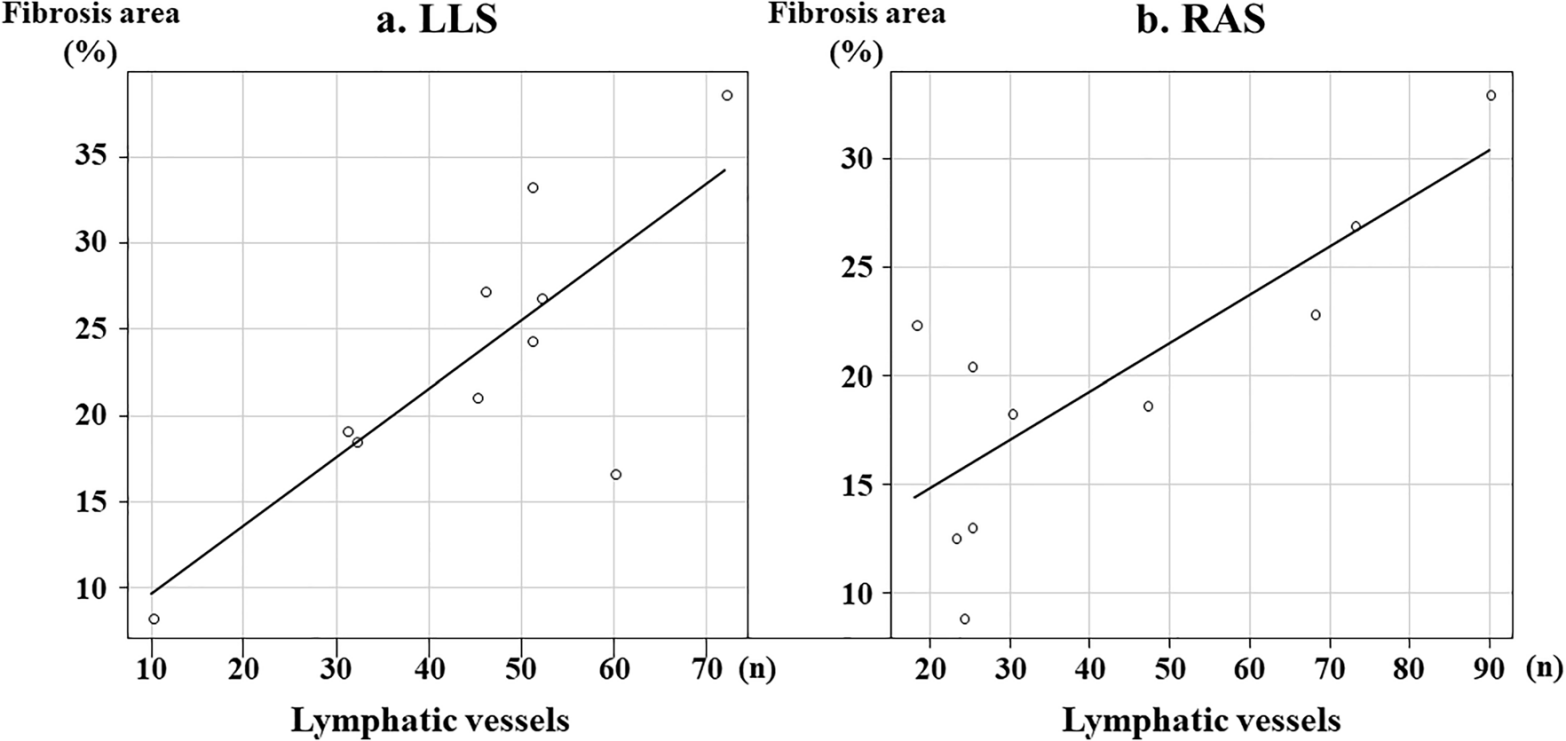
Correlation between the fibrosis area of all specimen areas and the number of lymphatic vessels in the LLS and RAS, separately. **a** LLS, left lateral segment; vertical axis, fibrotic area (%); horizontal axis, lymphatic vessels***.* b** RAS, right anterior segment; vertical axis, fibrotic area (%); horizontal axis, lymphatic vessels

**Fig. 5:**
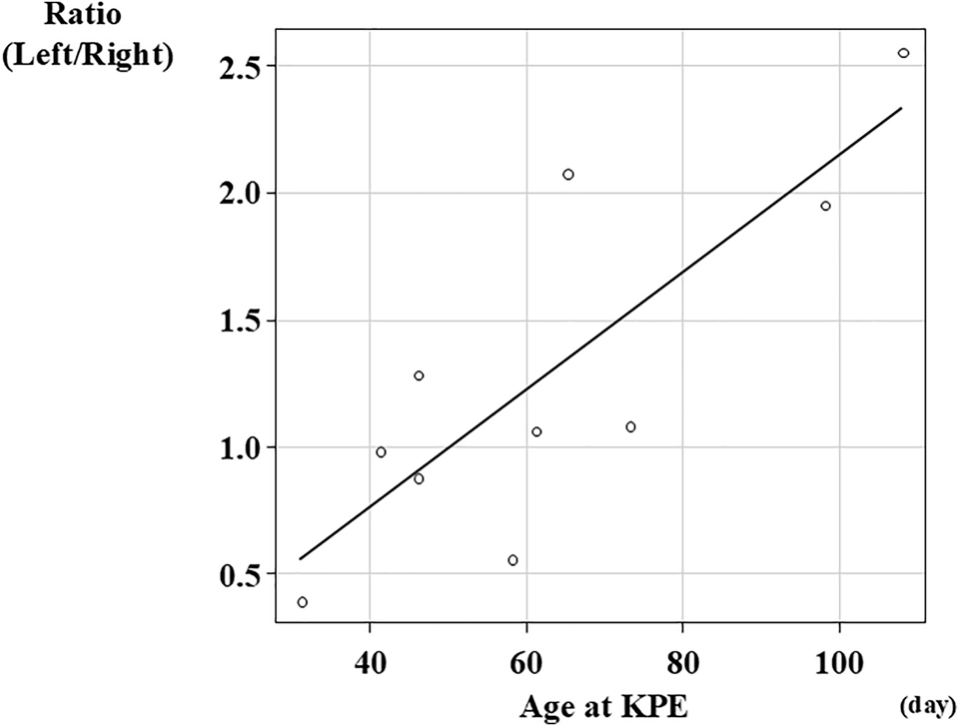
Correlation between age at KPE and the left-to-right ratio of the number of lymphatic vessels. Vertical axis, ratio (left/right); horizontal axis, age at KPE (day)

**Fig. 6:**
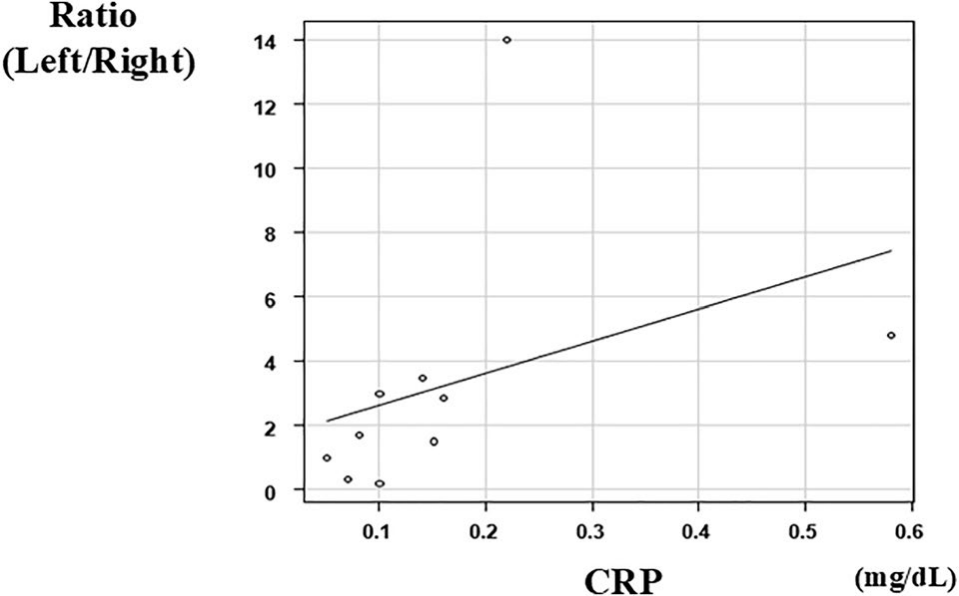
Correlation between pre-KPE CRP value and the left-to-right ratio of the number of lymphatic vessels with lumens. Vertical axis, lymphatic vessels ratio (left/right) without lumen; horizontal axis, pre-KPE CRP value(mg/dL)

Representative findings of lymphatic vessel morphology in the biopsy liver specimens are shown in Fig. 3. Histopathological findings in non-BA patients showed that lymphatic vessels are usually localized in the portal area (Fig. 3a). The histopathological findings of LLS (Fig. 3b) and RAS (Fig. 3c) in BA patients indicate that lymphatic capillaries proliferated into porto-portal bridging fibrosis. The correlation between the fibrotic area of all specimen areas and the number of lymphatic vessels is shown separately in the LLS and RAS in Fig. 4. This result showed a positive correlation between LLS (*r* = 0.571, *p* = 0.084) and RAS (*r* = 0.681, *p* = 0.030). The correlation between the age at KPE and the left-to-right ratio of the number of lymphatic vessels is shown in Fig. 5. Additionally, the correlation between pre-KPE CRP value and the left-to-right ratio of the number of lymphatic vessels with lumen is shown in Fig. 6. These results showed a significantly positive correlation (*r* = 0.723, *p* = 0.018) and revealed that the LLS/RAS lymphatic vessel ratio increases with an older age at KPE and with the pre-KPE CRP value.

## Discussion

This histopathological study unexpectedly showed no difference in the portal vein and lymphatic vascular morphology. The overgrowth of lymphatic capillaries is correlated with the age of KPE and the degree of fibrosis. The hepatic lymphatic vascular system has not been explored, and the mechanism of hepatic lymphangiogenesis remains largely unknown [5]. As a matter of fact, there have been no previous studies on lymphangiogenesis in pediatric liver diseases.

Adaptive immune responses are initiated by the migration of immune cells to inflamed sites and transmigration through lymphatic vessels to the lymph nodes to present antigens to T cells. Immune cells not only migrate through the lymphatic vessels but they also interact with the lymphatic vessels and promote lymphangiogenesis. The vascular endothelial growth factor receptor (VEGF)-C/VEGFR-3 axis is considered to be the most potent signaling pathway that regulates lymphangiogenesis. Lymphatic vessel dysfunction plays an important role in the pathogenesis of several diseases. Lymphangiogenesis, which is associated with increased lymphatic fluid in the BA liver, seems to be either due to an increased hydrostatic pressure associated with hepatic fibrosis or an ongoing postnatal immune response to antigens exposed during the neonatal period or both, thus leading to progressive bile duct obstruction, which is why the stool color changes gradually from a yellowish color to a gray color. Since there was no difference in the portal vein morphology between the LLS and RAS at the time of KPE, it is purported that the portal vein malformation prenatally occurs with the bile duct development during an early ductal plate phase [6, 7].

We also compared the preoperative CRP levels, which varied from 0.05 to 0.58 md/dl. The recent emphasis in cardiovascular medicine such as predictive associations with coronary events on “high-sensitivity” or “highly sensitive” CRP (hs-CRP) refers simply to the lower detection limit of the assay procedures being used [8]. Very sensitive CRP assays show non-specific inflammatory conditions, but they may play an important role in the pathogenesis of BA because CRP production is enhanced by the cytokine IL-6, which also drives T-helper 17 differentiation with diminished regulatory T cells in BA [9]. We previously reported that the CRP levels measured on postoperative day 57 predicted the surgical outcome of KPE [10]. In this series, significant lymphangiogenesis on the LLS compared to the RAS correlated with the preoperative CRP levels. This implies that the ongoing inflammatory process occurs postnatally and predominantly in the LLS before KPE. The HLF scores reflect the degree of liver damage. We found that the HLF score of the LLS was higher than or equal to that of the RAS in each patient. As the severity of HLF was reported to correlate with the aspartate aminotransferase level at postoperative one month [3], immune dysregulation in BA thus appears to become aggravated after birth and it even continues to progress after KPE in some cases.

Taken together, lymphangiogenesis in the BA liver seems to be closely related to the postnatal immune-mediated inflammatory process, which leads to more remarkable damage in the LLS. Among the various immune cells, CD68 + macrophages interact the most with lymphatic vessels and biliary epithelial cells with periportal IL-17A + cells [11] and M1 macrophages were reported to be associated with postoperative cholangitis and liver fibrosis and clinical outcome [12]. The cause of LLS atrophy is unknown at this time, but a least the viral infection theory seems to be unlikely. Future research on the degree of antigen presentation by dendritic cells or macrophages [13] is warranted to clarify the cause of the association between lymphangiogenesis and fibrosis of the portal tract and segmental atrophy. This might pave the way to eventually clarifying the etiopathogenesis of BA and may also help to develop new therapeutic strategies, such as by creating new ways to inhibit lymphangiogenesis.

## Limitations

There are several limitations associated with our findings in this study. First of all, the sample size was too small to evaluate morphological differences between the portal vein and lymphatic vessels in the LLS and RAS. The follow-up period of the patients was also rather short in this series; therefore, the prognostic significance of our findings might still be relatively obscure.

## Conclusions

Lymphangiogenesis on the LLS compared to the RAS was significantly correlated with the degree of fibrosis and the age at KPE. Whereas the PV vasculature showed no segmental difference, thus suggesting that postnatal immune-mediated damage is responsible for LLS atrophy.

## Data Availability

The datasets generated and/or analyzed during the current study are available from the corresponding author upon reasonable request.

## Abbreviations

BA: Biliary atresia
LLS: Left lateral segment
PV: Portal vein
KPE: Kasai portoenterostomy
RAS: Right anterior segment
WBC: White blood cell
CRP: C-reactive protein
d-bil: Direct bilirubin
ALT: Alanine aminotransferase
γ-GTP: γ-Glutamyl transpeptidase
FFPE: Fixed in formalin and embedded in paraffin
EMT: Elastica-Masson Trichrome
HLF: Hepatitis-like findings
LT: Liver transplantation
NLS: Native liver survivors
VEGF: Vascular endothelial growth factor
VEGFR: Vascular endothelial growth factor receptor
